# Treatment of Superficial Vein Thrombosis: Recent Advances, Unmet Needs and Future Directions

**DOI:** 10.3390/healthcare12151517

**Published:** 2024-07-31

**Authors:** Marcello Di Nisio, Giuseppe Camporese, Pierpaolo Di Micco, Romeo Martini, Walter Ageno, Paolo Prandoni

**Affiliations:** 1 Department of Medicine and Ageing Sciences, “G D’Annunzio” University, 66013 Chieti, Italy; marcello.dinisio@unich.it; 2 Clinica Medica 1, Department of Medicine, DIMED, Padua University Hospital, 35128 Padua, Italy; giuseppe.camporese@aopd.veneto.it; 3 AFO Medica, UOC Medicina Interna, P.O. Santa Maria delle Grazie, ASL Napoli 2 nord, 80076 Pozzuoli, Italy; pierpaolo.dimicco@aslnapoli2nord.it; 4 Unit of Vascular and Endovascular Surgery, San Martino Hospital, 32100 Belluno, Italy; 5 Dipartimento di Medicina Interna, Ospedale Regionale di Bellinzona e Valli Ente Ospedaliero Cantonale, 6500 Bellinzona, Switzerland; walter.ageno@eoc.ch; 6 Arianna Foundation on Anticoagulation, Via P. Fabbri 1/3, 40138 Bologna, Italy

**Keywords:** superficial vein thrombosis, deep vein thrombosis, pulmonary embolism, anticoagulation, fondaparinux, low-molecular-weight heparin, rivaroxaban

## Abstract

Once considered relatively benign, superficial vein thrombosis (SVT) of the lower limbs is linked to deep vein thrombosis (DVT) or pulmonary embolism (PE) in up to one fourth of cases. Treatment goals include alleviating local symptoms and preventing SVT from recurring or extending into DVT or PE. Fondaparinux 2.5 mg once daily for 45 days is the treatment of choice for most patients with SVT. Potential alternatives include intermediate-dose low-molecular-weight heparin or the direct oral factor Xa inhibitor rivaroxaban, however, these require further evidence. Despite these treatment options, significant gaps remain, including the role of systemic or topical anti-inflammatory agents alone or combined with anticoagulants, and the optimal duration of anticoagulation for patients at varying risk levels. Additionally, the efficacy and safety of factor Xa inhibitors other than rivaroxaban, management of upper extremity SVT, and optimal treatment for SVT near the sapheno-femoral or sapheno-popliteal junctions are not well understood. This narrative review aims to summarize current evidence on anticoagulant treatment for SVT, highlight key unmet needs in current approaches, and discuss how ongoing studies may address these gaps.

## 1. Introduction

Superficial vein thrombosis (SVT) refers to an inflammatory-thrombotic process involving a superficial vein, most often of the lower extremities, that usually presents with pain and a visible reddened, warm, tender cord along the vein. The diagnosis of SVT is made by compression venous ultrasonography, which can confirm the presence of the thrombus in the superficial vein and exclude thrombosis extension into the deep vein system.

Risk factors for SVT include varicose veins, central or peripheral venous catheters, surgery, trauma, cancer, pregnancy, oral contraceptives or hormonal replacement therapy, advanced age, autoimmune diseases, inherited thrombophilia, and a history of previous venous thrombosis.

The actual incidence of SVT is unclear. A French community-based study in an urban area with 265,687 adult residents reported an annual diagnosis rate of 0.64 per 1000 residents, increasing to 1.76 per 1000 among adults aged 75 or older, which could underestimate the actual rate as not all cases may be referred by general practitioners to vascular specialist and subsequently included in the study [1]. In a Dutch retrospective cohort study involving 140 primary care practices and approximately 240,000 patients, the SVT incidence was 1.31 per 1000 person-years, rising to 2.92 per 1000 person-years in those aged 80 or older [2]. Overall, SVT incidence appears similar to or higher than venous thromboembolism (VTE).

In a nationwide cohort study including all pregnant women who delivered between 1997 and 2017 in Denmark for a total of 1,276,046 deliveries, SVT incidence was 0.6 per 1000 person-years during the period between conception and 12 weeks postpartum [3]. SVT rates were relatively low during the first (0.1 per 1000 person-years), second (0.2 per 1000 person-years), and third trimester (0.5 per 1000 person-years) rising up to 1.6 per 1000 person-years during the post-partum period [3].

Though often seen as benign, SVT can extend, recur, or progress to deep vein thrombosis (DVT) or pulmonary embolism (PE) [1,4,5,6,7]. In the aforementioned French study, 24% of SVT patients had concomitant DVT, and 4.7% had concurrent PE, highlighting the importance of careful evaluation to exclude these conditions in all SVT patients [1]. A nationwide cohort study of 10,973 patients with a first-time SVT diagnosis reported a VTE incidence rate of 18.0 per 1000 person-years over a median follow-up of seven years [6]. VTE risk was highest in the first three months post-diagnosis, with incidences of 2.5% for DVT and 0.9% for PE, corresponding to 88-fold and 45-fold higher VTE risk compared to the general population. Consistent data from a French registry showed a VTE incidence of about 3–4% in the first three months post-SVT diagnosis [5]. Patients with isolated SVT had a similar recurrence risk (4–6%) to those with proximal DVT, although most events post-SVT occurred in the superficial vein system [4]. SVT involving the main trunk of the great saphenous vein (saphena magna) or small saphenous vein (saphena parva) and SVT near the sapheno-femoral or sapheno-popliteal junctions are considered to be at higher risk for progression to DVT or PE [4]. A recent retrospective study of 316 SVT patients, 30.7% of whom started anticoagulant treatment, found that 8.7% had concomitant asymptomatic DVT and 20.6% had SVT within 3 cm of the saphenofemoral or sapheno-popliteal junctions [7]. During the 3-month follow-up, 29.2% developed asymptomatic or symptomatic SVT extension, SVT recurrence, or VTE, with 82% of VTEs occurring in patients initially managed without anticoagulation [8]. In the study on pregnant women discussed above, the risk of developing VTE during the same pregnancy was considerable in women with antepartum SVT (hazard ratio 83.3, 95% CI 46.3 to 149.7) [3]. These epidemiological data warrant additional studies to specifically evaluate the efficacy and safety of SVT prophylaxis and treatment during pregnancy and the post-partum period.

Taken together, the currently available data suggest that the thrombotic risk in acute SVT patients is significant, and treatment should aim to prevent SVT extension, recurrence, or progression to VTE, in addition to alleviating local inflammation symptoms like pain and erythema. Various management approaches have been evaluated for SVT including anticoagulation, vein ligation or stripping, elastic stockings, and non-steroidal anti-inflammatory drugs (NSAIDs). Most studies evaluated the clinical course and management of SVT of the saphenous veins with or without concomitant involvement of varicosities, whereas trials specifically focused on isolated varicothrombosis are lacking.

This review focuses on the anticoagulant treatment for SVT, emphasizing key unmet needs with current options, and discussing the potential of ongoing studies to address current knowledge gaps.

## 2. Anticoagulant Treatment for SVT

A systematic review and meta-analysis of randomized controlled trials involving 7296 patients with lower extremity SVT summarized the efficacy and safety of topical, surgical, and medical treatments for SVT [9]. Treatment modalities included anticoagulation with low molecular weight heparin (LMWH), unfractionated heparin (UFH), NSAIDs, fondaparinux, and rivaroxaban; compression stockings; topical, intramuscular, or intravenous treatments; and surgical interventions like thrombectomy or ligation. Topical treatments improved local symptoms compared to placebo but lacked data on VTE and SVT extension. Similarly, evidence on surgical treatment was limited by study quality and poor reporting on thrombotic outcomes. Overall, data on topical or surgical management options were too sparse to guide clinical practice.

The Table 1 summarizes the principal characteristics of the main randomized clinical trials that evaluated anticoagulant treatment for SVT of the lower extremities.

Most evidence on anticoagulant treatment for SVT comes from the double-blinded, placebo-controlled CALISTO trial, which randomized 3002 participants with lower extremity SVT ≥ 5 cm in length to placebo or fondaparinux 2.5 mg subcutaneously once daily for 45 days [10]. Fondaparinux significantly reduced the incidence of the composite outcome of all-cause death, symptomatic PE, symptomatic DVT, or symptomatic SVT extension or recurrence by 85% at day 47, translating to a number needed to treat of 20. It also reduced symptomatic DVT or PE by 85%, SVT extension by 92%, and SVT recurrence by 79% without increasing major bleeding risk (0.1% in each group), clinically relevant non-major bleeding, minor bleeding, or total bleeding.

In the SURPRISE trial, the same fondaparinux regimen was compared with rivaroxaban 10 mg once daily for 45 days in 472 patients with SVT of at least 5 cm in length who were at high-risk defined by factors such as age over 65, male sex, previous SVT, DVT or PE, active cancer or cancer history, autoimmune disease, or non-varicose vein involvement [11]. Fondaparinux was associated with a non-significant reduction in symptomatic VTE (relative risk [RR] 0.33, 95% confidence intervals [CIs] 0.03 to 3.18) and clinically relevant non-major bleeding (RR 0.17, 95% CI 0.02 to 1.38), necessitating further evaluation in larger randomized studies. Neither group experienced major bleeding complications.

In a randomized, placebo-controlled trial, which was prematurely interrupted due to slow recruitment, patients with symptomatic lower extremity SVT of at least 5 cm length were randomized to 45 days of rivaroxaban 10 mg daily (n = 43) or placebo (n = 42) and followed-up for a total of 90 days [12]. Treatment failure, defined as the need for a non-study anticoagulant, occurrence of proximal DVT or PE, or surgery for SVT was observed in one rivaroxaban and five placebo patients (absolute risk reduction 9.0%, 95% CI −22 to 5.9%). There were no major bleeding events or deaths in either group. The authors found no difference in venous disease-specific or general health-related quality of life over 45 days. Rivaroxaban is the only direct oral factor Xa inhibitor evaluated for acute SVT treatment. No studies have assessed the direct thrombin inhibitor dabigatran in this context.

Several prospective cohort and randomized studies evaluated LMWH for SVT. In the Prospective Observational Superficial Thrombophlebitis (POST) cohort study, which included 844 patients with symptomatic SVT of the lower limbs ≥ 5 cm in length, 10.2% developed thromboembolic complications at three months (2.8% DVT, 0.5% PE, 3.3% SVT extension, or 1.9% SVT recurrence) despite 90.5% receiving anticoagulants, mostly LMWH for a median of 11 days [13]. In the STENOX study, the only randomized trial on LMWH using placebo as a control, both prophylactic and therapeutic doses of LMWH given for 8–12 days significantly reduced SVT extension or recurrence (prophylactic LMWH vs. placebo: RR 0.44, 95% CI 0.26 to 0.74; therapeutic LMWH vs. placebo: RR 0.46, 95% CI 0.27 to 0.77) [14]. LMWH was associated with a numerically lower VTE rate at the end of treatment, but differences with placebo diminished at three months. An indirect comparison between prophylactic LMWH and NSAIDs suggested a non-significant VTE reduction shortly after treatment (RR 0.45, 95% CI 0.04 to 4.89). These data should be interpreted cautiously due to indirect comparisons and small study size. The potential benefits of combining anticoagulants with topical or oral NSAIDs remain unexplored [9].

The POST and STEFLUX studies indicated that short-course LMWH may not sufficiently prevent thrombotic complications post-SVT, prompting evaluations of longer LMWH treatment durations. In the VESALIO study, 164 hospitalized or ambulatory SVT patients were randomized to therapeutic dose LMWH for 10 days followed by intermediate-dose LMWH for 20 days or prophylactic dose LMWH for 30 days [15]. While the incidence of the composite outcome, including SVT extension or recurrence, or VTE, was similar at 3 months, the rate of VTE during the treatment period was roughly half with therapeutic LMWH compared to the prophylactic dose (33% vs. 77%).

In the STEFLUX study, three regimens of LMWH were compared head-to-head in over 600 consecutive outpatients with SVT: 30-day intermediate dose LMWH, 30-day prophylactic dose LMWH, and 10-day intermediate dose LMWH [16]. At the 3-month follow-up, the 30-day intermediate dose LMWH was associated with a similar incidence of symptomatic VTE and recurrent SVT, but lower SVT extension compared to the 30-day prophylactic dose LMWH. The 30-day intermediate dose of LMWH reduced symptomatic VTE and SVT extension at 3 months, with a similar rate of SVT recurrence compared to the 10-day intermediate dose. There was no difference in DVT at 3 months or symptomatic PE, which occurred in none of the 30-day intermediate dose LMWH recipients and in one patient in each of the other two groups. No cases of major bleeding or heparin-induced thrombocytopenia (HIT) were observed in either the STEFLUX or VESALIO studies.

**Table 1:** Main randomized trials evaluating anticoagulant treatment for SVT of the lower extremities.

	Study Design	Number of Patients	Main Inclusion Criteria	Interventions	Primary Efficacy Outcome	Primary Safety Outcome
CALISTO [10]	Multicenter, randomized, double-blind, placebo-controlled	3002	Hospitalized or non-hospitalized patients with acute, symptomatic SVT ≥ 5 cm	Subcutaneous fondaparinux 2.5 mg once daily for 45 days Placebo for 45 days	Composite of death from any cause or symptomatic PE, symptomatic DVT, or symptomatic extension to the saphenofemoral junction or symptomatic recurrence of SVT at day 47	Major bleeding
SURPRISE [11]	Open-label, masked endpoint, randomized, non-inferiority phase 3b	472	Patients with symptomatic SVT ≥ 5 cm in a supra-genual superficial-vein segment and ≥1 additional thrombotic risk factor (aged ≥65 years, male sex, previous VTE, cancer, autoimmune disease, thrombosis of non-varicose veins)	Oral rivaroxaban 10 mg once daily for 45 days Subcutaneous fondaparinux 2.5 mg once daily for 45 days	Composite of symptomatic DVT or PE, progression or recurrence of SVT, and all-cause mortality at 45 days	Major bleeding
STENOX [14]	Multicenter, placebo-controlled	427	Hospitalized or non-hospitalized patients with SVT ≥ 5 cm	Subcutaneous LMWH (enoxaparin 40 mg once daily) for 8–12 days Subcutaneous LMWH (enoxaparin 1.5 mg/kg sc once daily) for 8–12 days Oral tenoxicam 20 mg once daily for 8–12 days Placebo for 8–12 day	Symptomatic PE and symptomatic and asymptomatic DVT at 12 days	Major bleeding
STEFLUX [16]	Multicenter, randomized, double-blind, placebo-controlled	664	Outpatients with SVT ≥ 4 cm involving internal or external saphenous veins or their collaterals	Intermediate-dose LMWH (parnaparin 8500 IU once daily) for 10 days followed by placebo for 20 days Intermediate-dose LMWH (parnaparin 8500 IU once daily for 10 days followed by 6400 IU once daily for 20 days) for 30 days Prophylactic-dose LMWH (parnaparin 4250 IU once daily) for 30 days	Composite of symptomatic and asymptomatic DVT, symptomatic PE and symptomatic or asymptomatic SVT recurrence at 33 days	Major bleeding
VESALIO [15]	Multicenter, double-blind, double-dummy	164	Hospitalized or non-hospitalized patients with SVT of internal saphenous vein with thrombosis extending up to 3 cm from the saphenous-femoral junction	Weight-adjusted LMWH (nadroparin full dose for 10 days followed by half dose for 20 additional days) for 30 days Fixed-dose LMWH (nadroparin 2850 anti-Xa IU) for 30 days	Composite of asymptomatic and symptomatic extension of SVT or VTE at 3 months	Major bleeding

DVT = deep vein thrombosis; LMWH = low-molecular-weight heparin; PE = pulmonary embolism; SVT = superficial vein thrombosis; VTE = venous thromboembolism.

Overall, the results of these studies suggest that intermediate or therapeutic doses of LMWH may be more effective than prophylactic doses, and that a course of at least one month is more effective than shorter treatment durations. However, the lack of a placebo control arm, the limited size and methodological weaknesses of the studies, and the substantial heterogeneity of the LMWH regimens evaluated represent significant limitations to firmly establish the efficacy and safety of LMWH and determine the optimal dose and duration of treatment. Nonetheless, leveraging the extensive experience with LMWH in patients with VTE, current guidelines suggest LMWH as a valid option for the treatment of SVT [17,18].

## 3. SVT Extending in Proximity to the Sapheno-Femoral or Sapheno-Popliteal Junctions

Patients with SVT in whom the thrombus head extends within 3 cm of the sapheno-femoral or sapheno-popliteal junctions are generally considered to be at higher risk of thromboembolic complications compared to patients with more distal SVT. These patients were excluded from trials on DVT treatment because their thrombosis did not involve the deep vein system. They were also excluded from studies on SVT treatment because their thrombotic risk was considered similar to that of patients with DVT, thus higher than that of patients with more distal SVTs. Consequently, data about the management of patients with SVT within 3 cm of the junctions remain scant, and the recommendation to consider a similar treatment as for patients with DVT is largely based on expert consensus.

An important aspect to acknowledge is that the relevant distance of the SVT head from the junction remains arbitrary. These uncertainties may translate into a heterogeneous approach to peri-junctional SVTs in clinical practice, as shown in a survey of 191 angiologists, vascular surgeons, hematologists, and other specialists involved in SVT management [19]. While 89% considered SVT extending < 3 cm from the sapheno-femoral junction as DVT, only approximately half (57%) prescribed at least 3 months of anticoagulation, and one-third (29%) adapted treatment duration based on serial ultrasonography results.

In a recent systematic review of 10,111 patients evaluating predictors of thrombotic complications in SVT, the location of the thrombus head relative to the sapheno-femoral junction was poorly reported across studies, hindering precise estimation of its relevance for clot progression [20]. A post-hoc analysis of the CALISTO study found that the risk of subsequent DVT or PE in patients receiving placebo who had SVT extension was similar regardless of SVT distance > 3 cm or ≤3 cm from the sapheno-femoral junction [21]. Patients treated with fondaparinux had lower rates of SVT extension ≤ 3 cm from the sapheno-femoral junction (0.3% vs. 3.6%), and none of these cases developed VTE.

In a recent analysis of the Computerized Registry of Patients with Venous Thromboembolism (RIETE), which included 374 patients with SVT ≤ 3 cm from the sapheno-femoral junction, 60.7% received therapeutic doses of LMWH or fondaparinux followed by vitamin K antagonists or direct oral anticoagulants, while 39.3% received either prophylactic dose fondaparinux or intermediate-dose LMWH [22]. At 3 months follow-up, the use of anticoagulants at therapeutic doses was associated with numerically lower thromboembolic events (1.3% vs. 2.7%) and higher major or clinically relevant nonmajor bleeding complications (1.3% vs. 0.7%).

Overall, the evidence about the management of peri-junctional SVTs appears to be scarce with residual substantial uncertainty about the optimal dose and duration of anticoagulation for patients with peri-junctional SVTs.

## 4. Duration of Anticoagulant Treatment for SVT

Current clinical guidelines suggest anticoagulant treatment with fondaparinux for 45 days for most patients with SVT of the lower extremities [17,18]. Potential alternatives are represented by rivaroxaban 10 mg once daily or intermediate-dose LMWH. However, whether the same agents and regimens can be effectively and safely adopted regardless of SVT extension or underlying risk factors remains unclear.

In the SURPRISE trial, the incidence of thromboembolic complications seemed to increase after treatment with fondaparinux or rivaroxaban, possibly due to the higher thrombotic risk of these patients compared to those in the CALISTO trial [11]. In the TROLL study, involving 229 patients with SVT ≥ 5 cm in length, the incidence rates of VTE and recurrent isolated SVT after anticoagulant treatment discontinuation were 3.5 and 4.4 per 100 person-years, respectively [23]. Despite 74% receiving a median of 45 days of anticoagulant treatment, mostly rivaroxaban 10 mg daily, 4.6% and 6.5% developed VTE or isolated SVT.

A post-marketing cohort study of 978 outpatients with SVT treated with fondaparinux (75.2%) for a median of 34 days and LMWH/unfractionated heparin (13%) for a median of 19 days found that the incidence of VTE was 0.8% during fondaparinux treatment, increasing to 2.4% in the post-treatment period [24]. A similar trend was observed with heparin (2.4% vs. 3.1%). In the more recent INSIGHTS-SVT study, which included 1150 patients with acute isolated SVT, the incidence of the composite of symptomatic DVT or PE, SVT extension, or recurrence at 3 months was 5.8% [25]. Most patients (93.6%) received anticoagulant treatment, but nearly half (48.6%) were treated for less than 25 days. On multivariable analysis, treatment duration was inversely related to the risk of thrombosis, suggesting that some high-risk patients with SVT might benefit from longer treatment durations.

Overall, these studies suggested that subgroups of patients with SVT might remain at substantial risk of thrombotic complications after treatment discontinuation.

In the systematic review discussed above, several risk factors, including older age, male sex, history of VTE, cancer, and absence of varicose veins, emerged as potential predictors of thrombosis in patients with SVT [20]. However, the actual relevance of these predictors could not be established precisely due to methodological flaws and biases in the included studies. Similar limitations precluded any conclusion about the importance of SVT length on thrombotic risk. The ongoing “START2–Registry: Survey on Anticoagulated Patients—registry” is enrolling patients with SVT of the upper or lower extremities, with follow-up scheduled at 3 months after treatment discontinuation. This study aims to provide data on the contemporary incidence of thrombotic complications after initial anticoagulation and identify risk factors for thrombosis progression or recurrence, potentially allowing for individualized treatment duration for SVT.

In the ongoing multicenter, randomized, double-blind METRO (MEsoglycan for Secondary Prevention of Superficial Vein Thrombosis) study (ClinicalTrials.gov: NCT 03428711), 650 adult patients with acute SVT of at least 5 cm in length and more than 3 cm from the sapheno-femoral or sapheno-popliteal junctions will be randomized to receive either oral mesoglycan 50 mg twice daily or placebo for 12 months after an initial 45-day course of fondaparinux 2.5 mg once daily. The hypothesis of the METRO study is that mesoglycan could reduce the risk of thrombosis, through inhibitory effects on thrombin and factor Xa and improvement of the endothelial function. The main objective of the study is to demonstrate the superiority of mesoglycan over placebo with respect to the primary composite efficacy outcome of asymptomatic or symptomatic recurrent SVT, SVT extension, and VTE at 12 months. The primary safety outcome is major bleeding. Secondary outcomes include the individual components of the primary outcome, patient quality of life as evaluated by the VEINES/Sym-QoL, vein recanalization, and the development of vein reflux. An additional follow-up visit is scheduled at 24 months to provide insights into the long-term incidence of thrombotic complications and risk factors for thrombosis up to 2 years after SVT diagnosis. The results of the METRO study are expected in 2025.

## 5. SVT of the Upper Extremities

SVT is a relatively frequent problem in hospitalized patients with peripheral intravenous catheters used for the administration of parenteral treatment, nutrition, blood products and other supportive measures. Less frequently, SVT of the upper extremities is secondary to infections, inflammation, trauma, or cancer. SVTs of the upper extremities may be complicated by infection, which increases healthcare costs by prolonging hospitalization and requiring additional nursing time. Typically, the peripheral catheter is removed, and anti-inflammatory creams or gel preparations are applied to the inflamed area to mitigate local signs and symptoms. While topical treatment appears to be associated with clinical improvement compared to placebo or no treatment, its effects on vein recanalization, SVT recurrence, or progression have not been evaluated [26]. The risk of thrombosis recurrence or progression in patients with SVT of the upper extremities has not been extensively evaluated, but it is generally considered to be lower compared to SVT of the lower extremities. There are no studies that have evaluated the use of anticoagulation for SVT of the upper extremities, making the role of anticoagulant treatment in this setting uncertain.

## 6. Conclusions

Anticoagulation with fondaparinux for 45 days is generally recommended as first line treatment for most patients with SVT of the lower extremities, while intermediate-dose LMWH or rivaroxaban 10 mg once daily are suggested as reasonable alternatives [17,18]. However, several uncertainties and knowledge gaps remain, including the optimal dose of LMWH, the efficacy and safety of systemic or topical anti-inflammatory agents either alone or in combination with anticoagulants, and the ideal duration of anticoagulation in high-risk patients. Risk stratification tools could help identify patients with SVT at very low risk for thrombotic complications, who might benefit from shorter courses of or no anticoagulation, thereby avoiding unnecessary treatment burden and side effects. A more conservative approach could be evaluated, for example, in cases of thrombosis of varicose collaterals, SVT of less than 5 cm in length, or thrombosis of antecubital veins secondary to peripheral vein catheters which may have a lower risk of thrombosis extension or recurrence. Additionally, further studies are needed to determine whether SVT extending close to the sapheno-femoral or sapheno-popliteal junctions requires a similar therapeutic approach as DVT, or if lower doses or shorter courses of anticoagulation would suffice.

## Data Availability

No new data were created or analyzed in this study.
